# Protective effect of Tualang honey against cadmium-induced morphological abnormalities and oxidative stress in the ovary of rats

**DOI:** 10.1186/s12906-020-02960-1

**Published:** 2020-05-29

**Authors:** Siti Suraya Ruslee, Siti Sarah Mohamad Zaid, Ikmal Hisyam Bakrin, Yong Meng Goh, Noordin Mohamed Mustapha

**Affiliations:** 1 Department of Environment, Faculty of Forestry and Environment, Universiti Putra Malaysia, 43400 Serdang, Selangor Malaysia; 2 Department of Pathology, Faculty of Medicine and Health Sciences, Universiti Putra Malaysia, 43400 Serdang, Selangor Malaysia; 3 Department of Veterinary Preclinical Science, Faculty of Veterinary Medicine, Universiti Putra Malaysia, 43400 Serdang, Selangor Malaysia; 4 Department of Veterinary Pathology and Microbiology, Faculty of Veterinary Medicine, Universiti Putra Malaysia, 43400 Serdang, Selangor Malaysia

**Keywords:** Tualang honey, Cadmium, Ovary, Antioxidant, Oxidative stress

## Abstract

**Background:**

To investigate the protective effects of Tualang honey against the toxicity effects induced by cadmium (Cd) on the ovary.

**Methods:**

A total of 32 female Sprague Dawley rats were taken and randomly divided into four groups (*n* = 8). Throughout the experimental period of 6 weeks, negative control-NC (vehicle deionized water), positive control-CD (Cd at 5 mg/kg), Tualang honey followed by Cd exposure-TH (Tualang honey at 200 mg/kg and Cd at 5 mg/kg) and Tualang honey control-THC (Tualang honey at 200 mg/kg) groups, were administered orally on a daily basis.

**Results:**

Rats exposed to Cd were significantly higher in ovarian weight, number of antral and atretic follicles as compared to the NC group. The disruptive effects of Cd on ovarian follicles were associated with a disruption in gonadotropin hormones and decreases in follicular stimulating hormone (FSH) and luteinizing hormone (LH). Moreover, a significant formation of oxidative stress in ovarian Cd-exposed rats has been proven by increasing the level of lipid peroxidation products (malondialdehyde) and decreasing the levels of enzymatic antioxidant (catalase). Interestingly, a daily supplementation of high antioxidant agents such as Tualang honey in these animals, caused significant improvements in the histological changes. Additionally, less atretic follicles were observed, restoring the normal level of LH and FSH (*P* < 0.001), and normalizing the ovarian malondialdehyde (*P* < 0.05) and catalase levels in comparison with CD group (*P* < 0.05).

**Conclusions:**

Tualang honey has protective effects against Cd-induced ovarian toxicity by reducing morphological abnormalities, restoring the normal levels of gonadotropin hormones and stabilizing equilibrium levels of lipid peroxidation and antioxidant enzyme in ovaries of rats.

## Background

Cadmium (Cd) is recognized as one of the endocrine disrupting chemicals (EDCs) which could have adverse effects on reproductive health by interfering with hormonal functions [1–3]. The main route of Cd exposure occurs primarily through dietary sources (seafood, grains, vegetables), smoking cigarettes, and drinking water [4]. It has a long biological half-life of 20 to 30 years [5], low excretion rate from the body, and can accumulate in blood, kidneys, liver [6] and in the reproductive organs [7–9].

The exposure of Cd has been well documented to induce oxidative stress (OS) by producing excessive reactive oxygen species (ROS) in the body [10–12]. This can cause damage to cell structures, including lipids, membranes, proteins and nucleic acids. Toxic effects of Cd are associated with increased levels of lipid peroxidation, resulting in the alterations of antioxidant defense systems including enzymes such as catalase (CAT), and non-enzymatic molecules, whose function is to act against the toxic effects of the free radicals [13, 14]. Previous studies revealed that Cd could induce significant changes in ovarian and uterus morphology [15–17]. The toxic effects of Cd may disrupt the process of steroidogenesis, leading to ovarian function-failure, and promoting hemorrhage and necrosis of the ovary [18, 19]. This becomes a matter of great concern, especially with all the consequences of Cd toxicity that can lead to infertility, particularly among women. Therefore, an extensive study on the possible therapeutic approaches to prevent toxic effects of Cd on the reproductive system is necessary.

According to traditional belief, high antioxidant natural products such as Tualang honey and *Ficus deltoidea* can help in maintaining the reproductive health. Study by Zaid et al. (2018) showed that *Ficus deltoidea* has the ability to protect female reproductive system from toxicity effects of EDC, namely Bisphenol-A (BPA) [20]. The study found that antioxidant activity of *Ficus deltoidea* has the capability to improve the percentage of normal estrous cycle, normalize the gonadotropins and sex steroid hormone levels, and reduce the formation of atretic follicles in the ovary of rats [21]. Thus, based on this scientific evidence, selection of Tualang honey in protecting female reproductive system from toxicity effects of Cd is unquestionable. Tualang honey is produced by *Apis dorsata* bees, reported to have high antioxidant properties [22, 23], and known to contain high amounts of phenolic acids and flavonoids, which have strong free radical-scavenging activities [23, 24]. A study by Khalil et al. had found that there are six phenolic acids and five flavonoid compounds in Tualang honey [22]. The findings were in agreement with a clinical study on the reduction of oxidative stress levels in the reproductive system of postmenopausal women [25]. In vivo study has found that Tualang honey is capable in preventing uterine and vaginal atrophy in postmenopausal animal models [26], besides exhibiting improvements in normal estrous cycles while reducing the formation of atretic follicles [27]. It has also been reported to prevent osteoporosis [28] and reduce the toxic effects of BPA on the morphology, lipid peroxidation, protein and molecular changes of the uterus [29]. In another study, Tualang honey had protective effects against oxidative stress induced by streptozotocin in diabetic rats [30]. Antioxidant properties of honey could be the reason for these positive results. A further evaluation on the possible beneficial effects of honey demands studies to be done.

Thus, the aim of the present study is to investigate the toxic effects of Cd on ovarian follicular development, hormonal profile of luteinizing hormone (LH), follicular stimulating hormone (FSH), estrogen (E_2_), progesterone (P_4_), lipid peroxidation and antioxidant enzyme activities. Consequently, the protective effects of Tualang honey in these selected parameters is also investigated. In this study we used rats as a model as it has been widely used in investigation of implication of EDC on reproductive health. Moreover, the processes underlying the development and physiology of rat and human models are considered the same [31].

## Methods

### Tualang honey and cadmium

The Tualang honey used in this study was supplied by Federal Agricultural Marketing Authority (FAMA), Ministry of Agriculture and Agro-Based Industry, Malaysia. Tualang honey was selected in this study because it has the highest antioxidant activity compared to the other local Malaysian honey, such as pineapple honey, gelam honey and Indian forest honey [24]. While the cadmium chloride used was obtained from Sigma Aldrich, USA.

### Animals

Thirty-two female Sprague Dawley rats aged 30 days (135–145 g) were used in this study. The animals were obtained from the Animal Resources Unit, Faculty of Veterinary Medicine, Universiti Putra Malaysia. All experimental procedures were conducted in accordance with the protocols set by National Institute of Health (Guide for the Care and Use of Laboratory Animals) which were approved by the Animal Ethics Committee, Universiti Putra Malaysia (UPM/IACUC/AUP-R059/2017). The administration of the rats for 6 weeks was conducted at Comparative Medicine and Technology Unit (COMeT), Universiti Putra Malaysia. All of the animals were acclimated for 7 days and housed under standard conditions where the temperature was set to 24 ± 2 °C with a 12 h light – 12 h dark cycle in polypropylene rat cages (595*380*200 mm). They were fed with standard laboratory pellets (Altromin, Germany) and distilled water ad libitum*.*

### Experimental design

The rats were randomly divided into four groups (*n* = 8) and administered by oral gavage as follows:

- Negative Control (NC): Vehicle deionized water
- Positive Control (CD): Cd at 5 mg/kg body weight
- Tualang honey + Cadmium (TH): Tualang honey at 200 mg/kg body weight followed by Cd at 5 mg/kg body weight
- Tualang honey Control (THC): Tualang honey at 200 mg/kg body weight

**Fig. 1:**
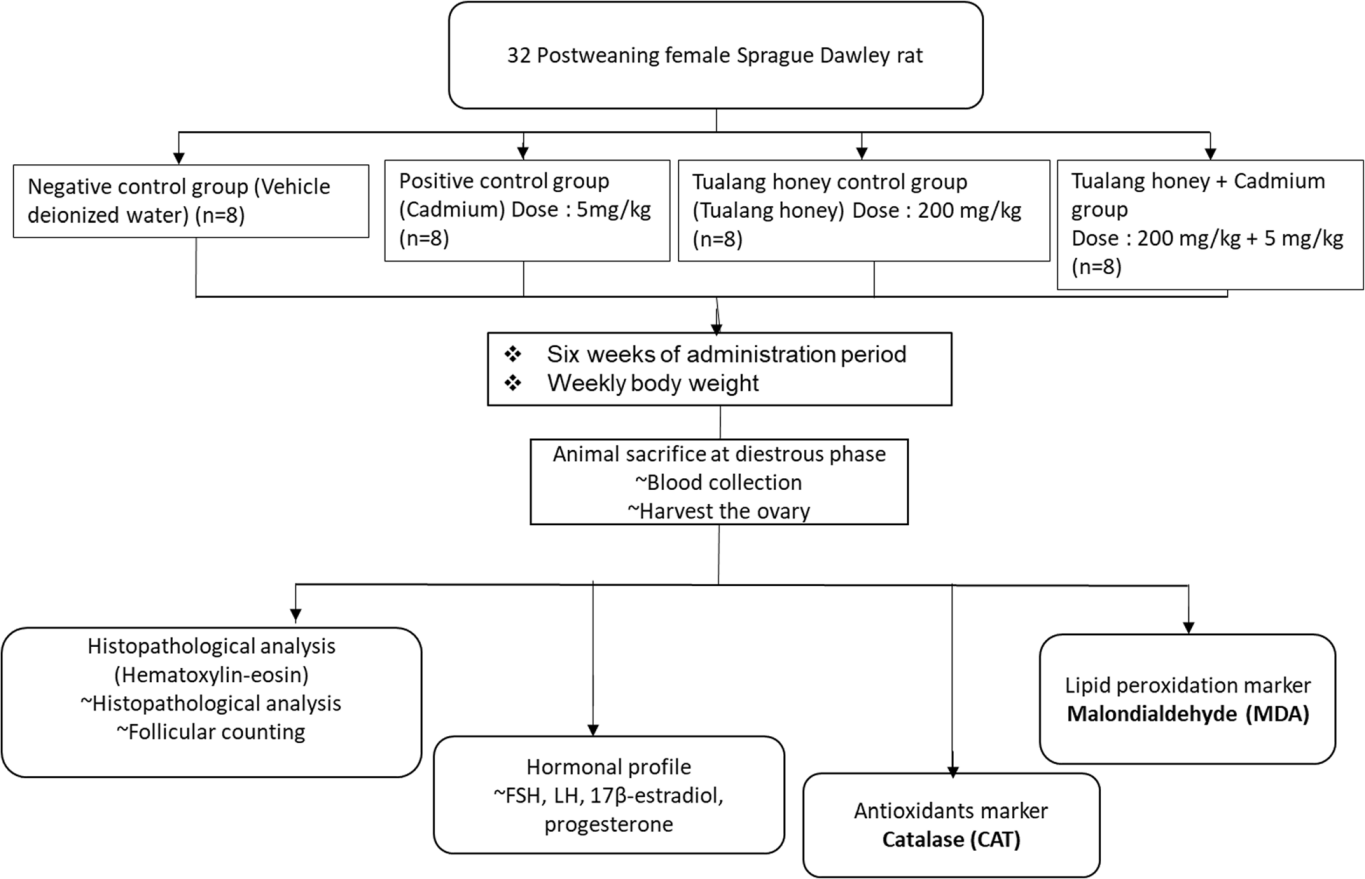
Illustration of the study design

This indicate that the cage is the experimental unit for comparing the treatment. The administration was done by oral gavage once daily in the morning for six consecutive weeks. Oral administration was done to imitate the most likely human exposure to cadmium. Tualang honey was freshly prepared daily to prevent oxidation of the antioxidants. The dose selection for Tualang honey was based on previous studies where there are significant beneficial effects of this natural product on female reproductive organ [26, 27]. The dose of 200 mg/kg is equivalent to one table spoon, which is the daily amount commonly consumed by an adult. The dose for Cd was based on previous studies, where it was shown to exhibit changes in morphological and biochemical parameters in the reproductive system [32]. Figure 1 show the illustration on the study design.

Throughout the administration period, the body weight of the rats was recorded weekly, started from day 1 of administration until the last day of administration. All rats were monitored once a day for health status. All rats were in good condition and no adverse events were observed. After 6 weeks of treatment, the rats were weighed and anesthetized by intraperitoneal injection of ketamine and xylazine (Ilium, Troy Laboratories Pty Limited, Australia) at doses of 50 and 10 mg/kg body weight [33], respectively. This method of anaesthesia has less effect on the rate of blood pressure. Subsequently, an approximately 5 ml of blood samples were collected from the abdominal aorta for hormonal profile analyses (LH, FSH, E_2_, P_4_). The rats were euthanized by cervical dislocation. The ovaries were removed by laparotomy and immediately weighed. The right side of the ovary was carefully dissected for histopathological examination, while the left side of the ovary, for oxidative stress markers and antioxidant level (catalase) analyses.

### Histopathological examination

The harvested ovary was fixed in 10% buffered formalin, hydrated in a graded series of ethanol, cleared by xylene and embedded in paraffin blocks. Each tissue block was sectioned into 5 μm thickness, stained with hematoxylin and eosin (Sigma-Aldrich, USA) and evaluated histologically using a light microscope attached with an image analyzer (Motic Images plus 2.0, Hong Kong). Sections were analyzed quantitatively and qualitatively. Classification and quantification of ovarian follicles were conducted according to the criteria as described in a previous study by Zhuang et al. [34]. The pathologist (IHB- 3rd author and NMM- 5th author) was blinded to the treatment received by the rats.

### Hormonal profile

The blood samples were collected from the abdominal aorta and put into a serum separator tube. After 2 h at room temperature, clotted blood samples were centrifuged at 1000 x g for 15 min. Extracted serum was stored at − 20 °C until subsequent analysis. Enzyme-linked immunosorbent assay (ELISA) (Cusabio, USA) was used for the measurement of the circulating levels of FSH, LH E_2_ and P_4_. Each sample was run in duplicate. In brief, 50 μl of each of the standards and serum samples were added to respective wells and incubated with 50 μl of HRP-conjugate enzyme and 50 μl antibody, for an hour at 37 °C. After the incubation period, aspirating and decanting steps were done three times using wash buffer solutions. Subsequently, 50 μl of Substrate A and Substrate B were added to each well and incubated again for another 15 min at 37 °C. Reactions were terminated using 50 μl of stop solution. The optical density (O.D) was measured at 450 nm using a microplate reader (Tecan, Switzerland). For determination of each hormonal level, a standard curve was constructed by plotting a graph of the absorbance reading against the concentration of the standards.

### Lipid peroxidation

The measurement of MDA level was carried out using an MDA kit (Cusabio, USA). The samples and the MDA standards were run in duplicate. In brief, the ovarian tissues were homogenized in phosphate-buffered saline (PBS) with the addition of butylated hydroxytoluene (BHT) solution (10 μl/mL sample volume) at 10,000 g on ice for 5 min. The supernatants were transferred into a new microcentrifuge tube for further analysis.

The supernatant (samples) were assayed directly for thiobarbituric acid reactive substances (TBARS) levels. Subsequently, 100 μl of the samples and the MDA standards were added into microcentrifuge tubes respectively. Afterwards, 100 μl of SDS lysis solution was added into each of the microcentrifuge tubes which were then incubated for 5 min at room temperature. After the incubation, 250 μl of thiobarbituric acid (TBA) was added before being re-incubated at 95 °C for 45–60 min. After cooling in an ice bath for 5 min, all the samples were centrifuged at 554 g for 15 min. The supernatants and the MDA standards (200 μl) were transferred into respective wells (microplates) and absorbance at 532 nm was measured. A standard curve was constructed for the determination of each MDA level, by plotting a graph of the absorbance reading against the concentration of the standards.

### Enzymatic antioxidant

Catalase (CAT) is an important antioxidant enzyme that is responsible for the degradation of the reactive oxygen species (ROS) and hydrogen peroxide [35]. A manual provided in the CAT kit was used as reference for the measurement of the CAT level (Cusabio, USA). Supernatant (from the ovarian samples that have been homogenized in PBS at 10,000 g on the ice for 5 min) and the catalase standards were added into respective wells (20 μL). Each sample and catalase standard were run in duplicate. Hydrogen peroxide working solution (50 μL) was directly added to each well and incubated at room temperature for 1 min. The reactions were terminated by using 50 μL of catalase quencher and mixed thoroughly. Subsequently, 5 μL of each well was transferred to a fresh well. Lastly, 250 μL of the chromogenic working solution was added to each well and the plate was incubated at room temperature for 40–60 min with vigorous mixing. The optical density was measured at 520 nm. A standard curve was plotted based on the absorbance reading and concentration of the standards.

### Sample size

Sample size of rats was based on previous studies [36, 37]. Thus, the sample size used in this study was eight rats in each group.

#### Statistical analysis

Data was evaluated using the Statistical Package for Social Sciences version 25.0 (SPSS Inc. Chicago, Illinois, USA) software. All of the data was presented as mean ± S.E.M. The normally distributed data was analyzed using one-way analysis of variance (ANOVA) followed by Bonferroni *post-hoc* test for multiple comparisons to identify significant differences between groups. The *P* value of less than 0.05 was considered significant.

## Results

### Body and ovary weight

The values of body weight gain, changes in body weight, absolute (wet weight) and relative weight of the ovary in all experimental groups are shown in Table 1. Even not statistically significant, the body weight gain and changes in body weight in the CD group were slightly lower than the NC group. Meanwhile, treatment of Tualang honey in Cd-exposed rats only caused slightly higher body weight compared to the rats in CD group. The changes in body weight in THC group were comparable to that of the NC group.

**Table 1:** Effects of Tualang honey on body and ovarian weights

Group	Body weight gain (g)	Changes in body weight gain (%)	Ovarian wet weight (mg)	Relative weight of ovary (wet weight/ body weight)
NC	117.76 ± 15.79	84.31 ± 7.41	86.60 ± 3.21	0.35 ± 0.01^bbb^
CD	97.65 ± 13.58	69.07 ± 6.37	102.80 ± 2.84	0.45 ± 0.02^aaa,c,dd^
TH	105.49 ± 14.30	76.50 ± 6.70	88.93 ± 3.64	0.38 ± 0.02^b^
THC	110.01 ± 14.71	79.42 ± 6.90	87.71 ± 3.44	0.37 ± 0.02^bb^

Data are expressed as mean ± SEM

1) ^aaa^*P* < 0.001 vs. NC

2) ^b^*P* < 0.05, ^bb^*P* < 0.01 and ^bbb^*P* < 0.001 vs. CD

3) ^c^*P* < 0.05 vs. TH

4) ^dd^*P* < 0.01 vs. THC

The ovary relative weight in the CD group was significantly higher than in the NC group. Interestingly, treatment with Tualang honey in Cd-exposed rats showed significant reduction compared to the Cd-exposed group without the treatment.

**Fig. 2:**
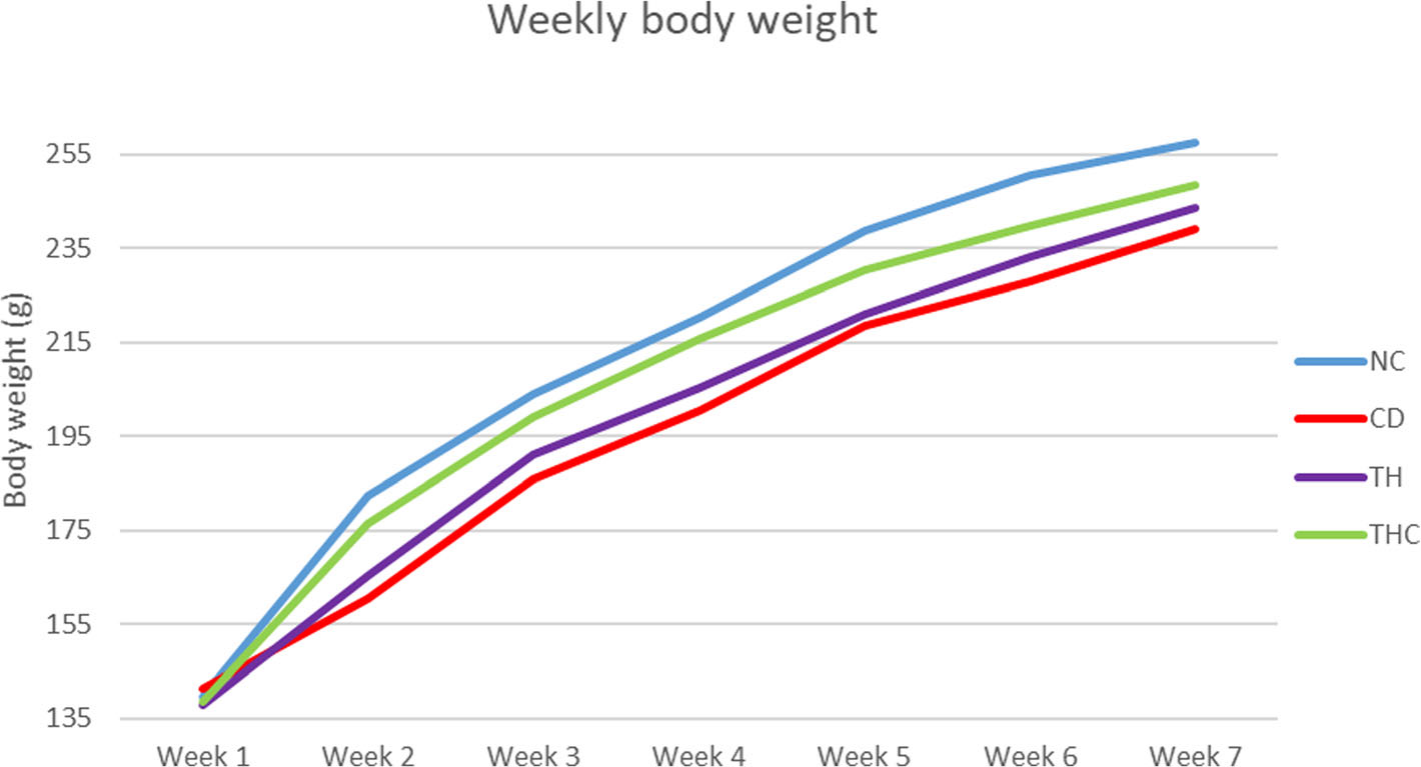
Weekly body weight growth curve of four experimental groups. NC (Negative Control) = Vehicle deionized water. CD (Positive Control) = Cd at 5 mg/kg body weight. TH (Tualang honey + Cadmium) = Tualang honey at 200 mg/kg body weight followed by Cd at 5 mg/kg body weight. THC (Tualang honey Control) = Tualang honey at 200 mg/kg body weight

All groups of rats showed a gradual increase in weekly body weight gain throughout the 6 weeks of treatment as shown in Fig. 2. During the period, rats in CD group showed only a slight increase in weekly body weight, especially compared to the NC and the THC group. Treatment with Tualang honey in Cd-exposed rats showed an improvement in body weight gain. However, the difference was not statistically significant among all the experimental groups.

### Follicular development

Generally, ovaries from all groups displayed all stages of follicular development as shown in Table 2.

**Table 2:** Effects of Tualang honey on the follicular ovarian development after 6 weeks of administration

Group	Primordial follicle	Primary follicle	Secondary follicle	Antral follicle	Corpora luteum	Atretic follicle
NC	6.00 ± 0.99	4.71 ± 0.54	4.16 ± 0.18	7.00 ± 0.55^bbb^	8.67 ± 1.03	9.43 ± 0.71^bb^
CD	4.50 ± 0.87	3.81 ± 0.39	3.95 ± 0.38	9.93 ± 0.50^aaa,c,dd^	7.95 ± 0.97	12.51 ± 0.83^aa,dd^
TH	5.00 ± 0.57	4.02 ± 0.44	4.00 ± 0.39	8.16 ± 0.55^b^	8.19 ± 0.89	11.00 ± 0.79
THC	5.90 ± 0.91	4.40 ± 0.48	4.14 ± 0.16	7.71 ± 0.37^bb^	8.19 ± 0.88	9.26 ± 0.86^bb^

Data are expressed as mean ± SEM

1) ^aa^*P* < 0.01 and ^aaa^*P* < 0.001 vs. NC

2) ^b^*P* < 0.05, ^bb^*P* < 0.01 and ^bbb^*P* < 0.001 vs. CD

3) ^c^*P* < 0.05 vs. TH

4) ^dd^*P* < 0.01 vs. THC

Quantitative results revealed no significant difference in preantral follicles (primordial, primary, secondary follicles) and corpora luteum number among all experimental groups. Even though not statistically different, the number of those follicles were lower in the CD group compared to the NC and the THC groups.

**Fig. 3:**
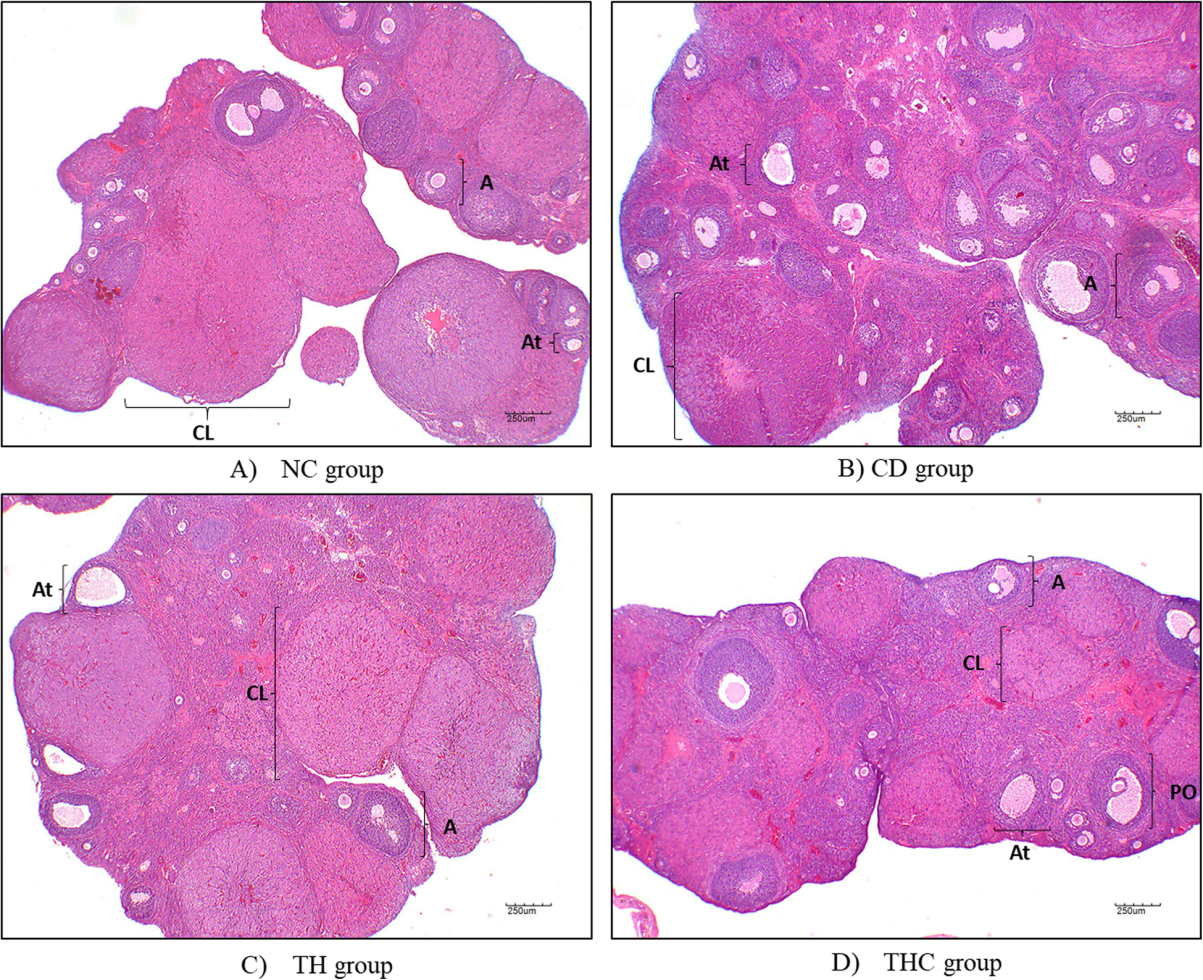
Representative ovarian section in all experimental groups. NC (Negative control) = vehicle deionized water; CD (Cadmium) = Cd at 5 mg/kg; TH (Tualang honey + Cadmium) = Tualang honey at 200 mg/kg followed by Cd at 5 mg/kg; THC (Tualang honey Control) = Tualang honey at 200 mg/kg. A: Antral; CL: Corpus luteum; At: Atretic; PO: Preovulatory. Scale bar = 250 μm

However, the number of antral and atretic follicles were statistically higher in the CD group as compared to the NC group. Follicles were counted based on histopathological examination (HPE) on the tissue in Fig. 3. The morphological abnormalities can be observed from Figure (B). Interestingly, treatment on Cd-exposed rats with Tualang honey showed less antral follicles and atretic follicles compared to the CD group.

### Hormonal profile

The FSH and LH hormones significantly reduced in the CD group compared to the NC group as shown in Table 3. However, FSH levels were significantly increased on treatment with Tualang honey in Cd-exposed rats (TH group). Meanwhile for LH serum, Tualang honey treatment on Cd-exposed rats showed only a slight increase compared to the CD group.

**Table 3:** Effects of Tualang honey on the hormonal profile of the rats after 6 weeks of administration

Group	Follicle Stimulating Hormone (FSH)	Luteinizing hormone (LH)	17β-Estradiol (E_2_)	Progesterone (P_4_)
NC	14.80 ± 0.24^bbb,ccc,ddd^	0.27 ± 0.03^bb,c^	3.52 ± 0.11^bbb,c^	53.29 ± 1.68^bbb,c^
CD	8.05 ± 0.13^aaa,ccc,ddd^	0.14 ± 0.03^aa^	4.16 ± 0.08^aaa^	42.35 ± 1.97^aaa,dd^
TH	9.81 ± 0.25^aaa,bbb,ddd^	0.19 ± 0.03^a^	3.96 ± 0.10^a^	46.10 ± 1.39^a^
THC	12.73 ± 0.35^aaa,bbb,ccc^	0.25 ± 0.02	3.76 ± 0.11	52.12 ± 1.55^bb^

Data were presented as mean ± SEM

1) ^a^*P* < 0.05, ^aa^*P* < 0.01 and ^aaa^*P* < 0.001 vs. NC

2) ^bb^*P* < 0.01 and ^bbb^*P* < 0.001 vs. CD

3) ^c^*P* < 0.05 and ^ccc^*P* < 0.001 vs. TH

4) ^dd^*P* < 0.01 and ^ddd^*P* < 0.001 vs. THC

For E_2_ serum, a significant increase was observed in the CD group compared to the NC group. However, only a slight reduction can be seen in the TH group for E_2_ serum. While for P_4_ serum, a significant reduction was observed in the CD group compared to the NC group. Treatment with Tualang honey in Cd-exposed rats showed a slight increase in P_4_ level compared to the CD group. Whereas, a significant decrease in P_4_ level was observed CD group as compared to the THC group.

### Biochemical analysis

Results on oxidative stress markers (MDA) and enzymatic antioxidant activity (CAT) are shown in Table 4. There were significant increases in lipid peroxidation, with significant reduction in the catalase activities in Cd-exposed rats (CD group) compared to the NC group. Interestingly, administration of Tualang honey in CD-exposed rats resulted in a significant reduction in lipid peroxidation and an increase in catalase activities.

**Table 4:** Effects of Tualang honey in lipid peroxidation and enzymatic antioxidant activities in the ovary from all experimental groups

Group	Malondialdehyde (MDA) (μmol/L)	Catalase (U/mL)
NC	1.28 ± 0.22 ^b^	294.40 ± 2.12 ^b^
CD	2.62 ± 0.32 ^a,c,d^	282.28 ± 2.54 ^a,c,ddd^
TH	1.41 ± 0.30 ^b^	293.15 ± 3.24 ^b^
THC	1.33 ± 0.26 ^b^	298.18 ± 2.20 ^bbb^

Data are expressed as Mean ± SEM

1) ^a^*P* < 0.05 vs. NC

2) ^b^*P* < 0.05 and ^bbb^*P* < 0.001 vs. CD

3) ^c^*P* < 0.05 vs. TH

4) ^d^*P* < 0.05 and ^ddd^*P* < 0.001 vs. THC

## Discussion

The 21st to 45th day after birth is the prepubertal phase (post-weaning) in rats. Based on the age comparison between rats and humans as proposed by Hood, the phase is equivalent to 2 to 12 year old in humans [38]. At this age, the function of hypothalamus-pituitary-gonadal axis is still unstable and the sex hormone levels are relatively low [39]. This will cause the child’s reproductive system to be highly vulnerable to xenoestrogenic substances, as compared to the adult. Children are primarily exposed to cadmium through oral exposure, by consuming cadmium contaminated food and beverages, inhaling tobacco smoke-polluted air, house dust and industrial pollution [40]. According to animal studies, early age exposure to cadmium may affect the hypothalamus-pituitary axis (HPA) functions, which can lead to endocrine disorders, thereby causing serious health effects which may become apparent at a later age [41].

In toxicological studies, assessments of body and organ weights are sensitive indicators for adverse effects of toxicants [42]. Several studies have reported that Cd exposure in rodent models correlates with weight gain [43–46]. In the present study, Cd caused a decrease in weight gain; however, it was not statistically different compared to the normal rats. A study by Sajjad et al. had found that there is a relation between loss of body weight and concentration of Cd in the hypothalamus and the pituitary gland in Cd-exposed animals [47]. This finding was probably due to the toxic effects of Cd on body systems which was associated with a high formation of free radicals [48]. It would lead to many cellular responses, thereby contributing to loss in body weight [49]. In contrast, Monsefi and Fereydouni, [50] and Nasiadek et al. [44] claimed that there are no significant effects of Cd administration on the body weight of the rats. Results were different from other studies, possibly due to differences in exposure route, dosage and rat strain used [15, 16, 51]. Thus, our findings are consistent with the later findings. However, results in our experiments could be significant if the exposure period of cadmium in rats was prolonged, something to look into in future experimental studies.

Cadmium can mimic the action of the endogenous estrogen and may interfere with endocrine functions [15, 52–55]. Previous studies have found that the pituitary gland is a highly sensitive organ to Cd exposure [56–59]. It has been recognized to alter prolactin secretion, by modifying the lactotroph cell activity in the pituitary gland, resulting in biochemical, genomic, and morphological changes [56]. Moreover, Cd is readily absorbed and accumulated in the pituitary gland of rats [60]. This could be attributed to the alteration in the normal function of HPA. In our study, the levels of FSH and LH serum were significantly lower in Cd-exposed rats as compared to the normal rats. The interference in the normal production of FSH and LH is an indicator of the interference of normal production of gonadotrophin releasing hormone (GnRH). This may cause disruption in the normal mechanism of HPA. Reduction in FSH levels may inhibit growth and maturation of immature oocytes into mature follicles before ovulation (primordial, primary and secondary follicles). This could consequently inhibit in later ovulation processes, reflecting in small amounts of corpus luteum. Consequently, corpus luteum deficiency would prevents P_4_ levels from rising. Low levels of LH serum prevent the ovulation process, which is attributed to the formation of large cystic antral follicles. The high number of large cystic antral follicles could be reflected in the higher ovarian weights in Cd-exposed rats compared to the normal control rats. Cadmium has been found to disrupt the hormonal function [61] and was able to inhibit the ovulation by interfering with the ovulatory surge of LH [62]. Interestingly, treatment of Tualang honey in Cd-exposed rats might contributes to a significant normalizing of gonadotropin hormone levels. Normalizing in LH levels will triggered ovulation and thereby attribute to the formation of corpus luteum for the production of P_4_. In summary, treatment of Tualang honey in Cd-exposed rats has normalized the positive and the negative feedback mechanisms in the HPA and ovary. As a result, number of the ovarian follicles would be normalized as control rats. Previous study found that quercetin and kaempferol that contained in Tualang honey exerted the free radical scavenging activity [24, 26, 63]. In addition, estrogenic properties of quercetin and kaempferol might compete with EDC to bind to estrogen receptors, thereby against metalloestrogens of Cd. Study by Cao et al. [64] showed a beneficial effect of these compounds in normalizing the mechanism of HPA. This is due to the structural similarity to the endogenous estrogen (17β-estradiol), which promotes estrogenic effects [65]. Therefore, it has the potential to compete with estrogen-like effects of Cd to bind to ERα of the hypothalamic-pituitary-gonadal axis, thereby stimulating GnRH release. This hormone is responsible for regulating the reproductive system, that acts on receptors in the anterior pituitary gland of the brain and signals the gonadotrophic cells to secrete LH and FSH serum. Thus, increase in FSH stimulate follicular growth and induce normal ovulation process with the collaboration of LH serum.

In our study, rats exposed to Cd were significantly high in the ovarian lipid peroxidation levels, and showed reducing enzymatic antioxidant activity compared to the normal rats. This might indicate that Cd acts as an endocrine disruptor that induces oxidative stress and could interrupt the endogenous antioxidant-oxidative stress equilibrium. Cadmium toxicity mechanisms include glutathione depletion and protein-bound sulfhydryl groups, resulting in the increased production of reactive oxygen species (ROS) and free radical formation such as superoxide anion, nitric oxide, hydrogen peroxide, and hydroxyl radicals [66–68]. The oxidative stress results in damages in lipid metabolites, DNA damage, altered gene expression and apoptosis [14, 69]. Furthermore, a study by Ciani et al. stated that the formation of atretic follicles (follicles that undergo atresia from various stages) is due to the accumulation of toxic metabolites resulting from oxidative stress [70]. This is reflected by high numbers of atretic follicles.

Antioxidant enzymes are an essential part of the cellular defense against ROS. The catalase (CAT) is an antioxidant enzyme function that reduces or scavenges hydrogen peroxide to form water and oxygen [71]. In this present study, rats exposed to Cd showed significantly lower ovarian CAT levels and were high in lipid peroxidation activity compared to the normal rats. The imbalance between ROS production and scavenging activities may induce oxidative stress which in turn can cause oxidative damage and alter many cellular functions, including a loss of enzymatic activity [72]. However, with the supplementation of Tualang honey in Cd-exposed rats, the activities of CAT were significantly restored. The antioxidant properties of Tualang honey could be the reason for the improvement in antioxidant enzymatic levels. When endogenous antioxidants are insufficient to eliminate ROS from the body, obtaining the exogenous natural antioxidants becomes important for the body. Many researchers notion that flavonoids and phenolic acids are responsible for the antioxidant activity of honey as it has the ability in scavenging the free radical formation [22, 23, 73]. Phenolic compounds work against oxidative stress as they have the properties of reducing agents (they have hydrogen- or electron-donating capacity) with chemical structure of hydroxyl groups [74, 75]. The more hydroxyl groups there are in phenolic compounds, the more efficiently they can react as antioxidant agents due to their ability to donate hydrogen atoms to free radicals. Thus, the free radical formation will be reduced. Besides, vitamin E and C contained in honey also has a protective effect against oxidative stress. Vitamin E helps to prevent lipid peroxidation reactions by inhibiting the production of lipid radicals in cellular membranes, whereas vitamin C is a water-soluble antioxidant that can interact directly against free radicals in cytosol and extracellular fluids to reduce oxidative damage [76]. Therefore, it is possible to propose that phenolic compounds and vitamin C and E in Tualang honey might help in the reduction of ROS and free radical formation, subsequently attributing to reduced lipid peroxidation and increased activities of CAT.

Apart from that, Tualang honey is rich in various nutrients, consists of complex sugars, amino acids, minerals, proteins, organic acids, vitamins and various phytochemicals that contribute to its antioxidant effects [22, 24]. Flavonols (quercetin and kaempferol) which are also contained in honey, are the most common and largest subgroup of flavonoids, and can also be obtained from fruits and vegetables [63]. Quercetin and kaempferol exerted the free radical scavenging activity and estrogenic properties which allow it to bind to estrogen receptors and combat against metalloestrogens like Cd. The increase in the levels of gonadotropins hormones (FSH and LH) observed in the group treated with Tualang honey in Cd-exposed rats, showed improvement in the normal function of the HPA. The HPA controls many body systems, including the endocrine system. The increase in FSH levels resulted in the increasing number of preantral follicle (primordial, primary and secondary) formation. A surge in LH levels triggers ovulation and is attributed to the development of corpus luteum. This leads to an increase in P_4_ and E_2_ levels. Additionally, treatment of Tualang honey in Cd exposed rats resulted in significant improvements in the morphological abnormalities with a reduction in the number of atretic follicles. More interestingly, the treatment of Tualang honey on Cd-exposed rats result in the reduction of lipid peroxidation and an increase in enzymatic antioxidant activity. These findings prove that Tualang honey has the ability of reducing ovarian toxicity induced by Cd.

## Conclusion

In conclusion, our results demonstrated that daily supplementation of Tualang honey has protective effects in reducing the ovarian toxicity induced by Cd. The protective effects may be observed in the form of a reduction in morphological abnormalities in the ovary, restoration of the gonadotropin hormones, reduction in the lipid peroxidation level and increase in the levels of enzymatic antioxidants.

## Data Availability

The datasets used and/or analyzed during the current study are available from the corresponding author upon reasonable request.

## Abbreviations

Cd: Cadmium
FSH: Follicular stimulating hormone
LH: Luteinizing hormone
E_2_: 17β-Estradiol
P_4_: Progesterone
EDC: Endocrine disrupting chemical
OS: Oxidative stress
ROS: Reactive oxygen species
CAT: Catalase
MDA: Malondialdehyde
NC: Negative control group
CD: Cadmium control group
TH: Tualang honey with cadmium group
THC: Tualang honey control group
ELISA: Enzyme-linked immunosorbent assay
O.D: Optical density
PBS: Phosphate buffered saline
BHT: Butylated hydroxytoluene
TBARS: Thiobarbituric acid reactive substance
TBA: Thiobarbituric acid
ANOVA: Analysis of variance
HPE: Histopathological examination
HPA: Hypothalamus pituitary axis
GnRH: Gonadotrophin releasing hormone
DNA: Deoxyribonucleic acid

## References

[1] Ciarrocca M, Capozzella A, Tomei F, Tomei G, Caciari T (2013). Exposure to cadmium in male urban and rural workers and effects on FSH, LH and testosterone. Chemosphere.

[2] Mendiola J, Moreno JM, Roca M, Vergara-Juárez N, Martínez-García MJ, García-Sánchez A (2011). Relationships between heavy metal concentrations in three different body fluids and male reproductive parameters: a pilot study. Environ Health.

[3] Zeng X, Jin T, Buchet JP, Jiang X, Kong Q, Ye T (2004). Impact of cadmium exposure on male sex hormones: a population-based study in China. Environ Res.

[4] Byrne C, Divekar SD, Storchan GB, Parodi DA, Martin MB (2009). Cadmium - A metallohormone?. Toxicol Appl Pharmacol.

[5] McElroy JA, Kruse RL, Guthrie J, Gangnon RE, Robertson JD (2017). Cadmium exposure and endometrial cancer risk: a large midwestern U.S. population-based case-control study. PLoS One.

[6] Bhattacharya MH, Wilson AK, Rajan SS, Jonath M. Biochemical pathways in cadmium toxicity. In: Zalups RK, Koropatnick J, editors. Molecular Biology and Toxicology of Metals. London: Taylor and Francis; 2000. pp. 34–74.

[7] Piasek M, Blanuša M, Kostial K, Laskey JW (2001). Placental cadmium and progesterone concentrations in cigarette smokers. Reprod Toxicol.

[8] Zadorozhnaja TD, Little RE, Mendel NA, Taylor RJ, Presley BJ, Galden BC (2000). Concentrations of arsenic, cadmium, copper, lead, mercury and zinc in human placentas from two cities in Ukraine. J Toxicol Environ Health.

[9] Varga B, Zsolnai B, Paksy K, Náray M, Ungváry GY (1993). Age dependent accumulation of cadmium in the human ovary. Reprod Toxicol.

[10] Cuypers A, Plusquin M, Remans T, Jozefczak M, Keunen E, Gielen H (2010). Cadmium stress: an oxidative challenge. BioMetals..

[11] Nwokocha CR, Nwokocha MI, Aneto I, Obi J, Udekweleze DC, Olatunde B (2012). Comparative analysis on the effect of *Lycopersicon esculentum* (tomato) in reducing cadmium, mercury and lead accumulation in liver. Food Chem Toxicol.

[12] Olaolu TD (2018). Effect of cadmium on female reproduction and treatment options. Res J Obstet Gynecol.

[13] Roopha DP, Padmalatha C (2012). Effect of herbal preparation on heavy metal (cadmium) induced antioxidant system in female Wistar rats. J Med Toxicol.

[14] Patra RC, Rautray AK, Swarup D (2011). Oxidative stress in lead and cadmium toxicity and its amelioration. Vet Med Int.

[15] Ali I, Damdimopoulou PPE, Mäkelä SI, Berglund M, Stenius U, Åkesson A (2010). Estrogen-like effects of cadmium *in vivo* do not appear to be mediated via the classical estrogen receptor transcriptional pathway. Environ Health Perspect.

[16] Höfer N, Diel P, Wittsiepe J, Wilhelm M, Degen GH (2009). Dose and route dependent hormonal activity of the metalloestrogen cadmium in the rat uterus. Toxicol Lett.

[17] Wang Y, Wang X, Wang Y, Fan R, Qiu C, Zhong S (2015). Effect of cadmium on cellular ultrastructure in mouse ovary. Ultrastruct Pathol.

[18] Rafati-Rahimzadeh M, Rafati-Rahimzadeh M, Kazemi S, Moghadamnia A (2017). Cadmium toxicity and treatment: an update. Casp J Intern Med.

[19] Thompson J, Bannigan J (2008). Cadmium: toxic effects on the reproductive system and the embryo. Reprod Toxicol.

[20] Zaid SSM, Othman S, Kassim NM (2018). Protective role of *Ficus deltoidea* against BPA-induced impairments of the follicular development, estrous cycle, gonadotropin and sex steroid hormones level of prepubertal rats. J Ovarian Res.

[21] Abrahim NN, Abdul-Rahman PS, Aminudin N (2018). The antioxidant activities, cytotoxic properties, and identification of water-soluble compounds of *Ficus deltoidea* leaves. PeerJ..

[22] Khalil MI, Alam N, Moniruzzaman M, Sulaiman SA, Gan SH (2011). Phenolic acid composition and antioxidant properties of Malaysian honeys. J Food Sci.

[23] Mohamed ZBH, Alfarisi HAH (2017). Tualang honey: composition, physiochemical properties and clinical importance. Int Res J Pharm.

[24] Kishore RK, Halim AS, Syazana MSN, Sirajudeen KNS (2011). Tualang honey has higher phenolic content and greater radical scavenging activity compared with other honey sources. Nutr Res.

[25] Shafin N, Othman Z, Zakaria R, Hussain NNH (2014). Tualang honey supplementation reduces blood oxidative stress levels/activities in postmenopausal women. ISRN Oxidative Med.

[26] Zaid SSM, Sulaiman SA, Sirajudeen KNM, Othman NH (2010). The effects of Tualang honey on female reproductive organs, tibia bone and hormonal profile in ovariectomised rats-animal model for menopause. BMC Complement Altern Med.

[27] Zaid SSM, Othman S, Kassim NM (2014). Potential protective effect of Tualang honey on BPA-induced ovarian toxicity in prepubertal rat. BMC Complement Altern Med.

[28] Zaid SSM, Sulaiman S, Othman N, Soelaiman I, Shuid A, Mohamad N (2012). Protective effects of Tualang honey on bone structure in experimental postmenopausal rats. Clinics..

[29] Zaid SSM, Kassim NM, Othman S (2015). Tualang honey protects against BPA-induced morphological abnormalities and disruption of ERα, ERβ, and C3 mRNA and protein expressions in the uterus of rats. Evid-Based Complement Altern Med.

[30] Omotayo EO, Gurtu S, Sulaiman SA, Wahab AMS, Sirajudeen KN, Salleh MS (2010). Hypoglycemic and antioxidant effects of honey supplementation in streptozotocin-induced diabetic rats. Int J Vitam Nutr Res.

[31] Auger J, Eustache F, Rouiller-Fabre V, Canivenc-Lavier MC, Livera G (2014). Integrative rodent models for assessing male reproductive toxicity of environmental endocrine active substances. Asian J Androl.

[32] Paksy K, Varga B, Lázár P (1996). Effect of cadmium on female fertility, pregnancy and postnatal development in the rat. Acta Physiol Hung.

[33] Hatzopoulos S, Petruccelli J, Laurell G, Finesso M, Martini A (2002). Evaluation of anesthesia effects in a rat animal model using otoacoustic emission protocols. Hear Res.

[34] Zhuang XL, Fu YC, Xu JJ, Kong XX, Chen ZG, Luo LL (2010). Effects of genistein on ovarian follicular development and ovarian life span in rats. Fitoterapia..

[35] Nandi A, Yan LJ, Jana CK, Das N (2019). Role of catalase in oxidative stress and age associated degenerative diseases. Oxidative Med Cell Longev.

[36] Saki G, Jasemi M, Sarkaki A, Fathollahi A (2013). Effect of administration of vitamins C and E on fertilization capacity of rats exposed to noise stress. Noise Health.

[37] Nadri F, Khavanin A, Mazaheri Z, Khajehnasiri F (2018). The effect of noise stress on adult male rat sperm parameters and the protective effect of hydroalcoholic *Cinnamomum verum* extract: an experimental study. Iran Red Crescent Med J.

[38] Hood RD (2005). Developmental and reproductive toxicology: a practical approach.

[39] Li Y, Zhang W, Liu J, Wang W, Li H, Zhu J (2014). Prepubertal bisphenol a exposure interferes with ovarian follicle development and its relevant gene expression. Reprod Toxicol.

[40] Schoeters G, Hond DE, Zuurbier M, Naginiene R, Hazel VDP, Stilianakis N (2006). Cadmium and children: exposure and health effects. Acta Paediatr.

[41] Schantz SL, Widholm JJ (2001). Cognitive effects of endocrine-disrupting chemicals in animals. Environ Health Perspect.

[42] Hood RD. Handbook of developmental toxicology. Boca Raton: CRC Press; 1996. p. 790.

[43] Barański B, Sitarek K (1987). Effect of oral and inhalation exposure to cadmium on the oestrous cycle in rats. Toxicol Lett.

[44] Nasiadek M, Danilewicz M, Sitarek K, Świątkowska E, Daragó A, Stragierowicz J (2018). The effect of repeated cadmium oral exposure on the level of sex hormones, estrous cyclicity, and endometrium morphometry in female rats. Environ Sci Pollut Res.

[45] Pond WG, Walker EF (1975). Effect of dietary Ca and cd level of pregnant rats on reproduction and on dam and progeny tissue mineral concentrations. Exp Biol Med.

[46] Sutou S, Yamamoto K, Sendota H, Sugiyama M (1980). Toxicity, fertility, teratogenicity, and dominant lethal tests in rats administered cadmium subchronically. Ecotoxicol Environ Saf.

[47] Sajjad S, Malik H, Farooq U, Rashid F, Nasim H, Tariq S (2014). Cadmium chloride toxicity revisited: effect on certain andrological, endocrinological and biochemical parameters of adult male rabbits. Physiol Res.

[48] Waisberg M, Joseph P, Hale B, Beyersmann D (2003). Molecular and cellular mechanisms of cadmium carcinogenesis. Toxicology..

[49] Jamakala O, Rani U (2015). Amelioration effect of zinc and iron supplementation on selected oxidative stress enzymes in liver and kidney of cadmium-treated male albino rat. Toxicol Int.

[50] Monsefi M, Fereydouni B (2013). The effects of cadmium pollution on female rat reproductive system. J Infertil Reprod Biol.

[51] Liu J, Huang H, Zhang W, Li H (2010). Cadmium-induced increase in uterine wet weight and its mechanism. Birth Defects Res B Dev Reprod Toxicol.

[52] Fechner P, Damdimopoulou P, Gauglitz G (2011). Biosensors paving the way to understanding the interaction between cadmium and the estrogen receptor alpha. PLoS One.

[53] Iavicoli I, Fontana L, Bergamaschi A (2009). The effects of metals as endocrine disruptors. J Toxicol Environ Health B Crit Rev.

[54] Kluxen FM, Höfer N, Kretzschmar G, Degen GH, Diel P (2012). Cadmium modulates expression of aryl hydrocarbon receptor-associated genes in rat uterus by interaction with the estrogen receptor. Arch Toxicol.

[55] Stoica A, Katzenellenbogen BS, Martin MB (2000). Activation of estrogen receptor-α by the heavy metal cadmium. Mol Endocrinol.

[56] Calderoni AM, Biaggio V, Acosta M, Oliveros L, Mohamed F, Giménez MS (2010). Cadmium exposure modifies lactotrophs activity associated to genomic and morphological changes in rat pituitary anterior lobe. BioMetals..

[57] Jiménez-Ortega V, Barquilla CP, Fernández-Mateos P, Cardinali DP, Esquifino AI (2012). Cadmium as an endocrine disruptor: correlation with anterior pituitary redox and circadian clock mechanisms and prevention by melatonin. Free Radic Biol Med.

[58] Lafuente A, Esquifino AI (1999). Cadmium effects on hypothalamic activity and pituitary hormone secretion in the male. Toxicol Lett.

[59] Miler EA, Nudler SI, Quinteros FA, Cabilla JP, Ronchetti SA, Duvilanski BH (2010). Cadmium induced-oxidative stress in pituitary gland is reversed by removing the contamination source. Hum Exp Toxicol.

[60] Kollmer WE (1980). Uptake and retention of cadmium-109 in the pituitary, the adrenals and the thyroid of the laboratory rat. Int J Appl Radiat Isot.

[61] Pollack AZ, Ranasinghe S, Sjaarda LA, Mumford SL (2014). Cadmium and reproductive health in women: a systematic review of the epidemiologic evidence. Curr Environ Health Rep.

[62] Saksena SK, Salmonsen R (1983). Effects of cadmium chloride on ovulation and on induction of sterility in the female golden hamster. Biol Reprod.

[63] Panche AN, Diwan AD, Chandra SR (2016). Flavonoids: an overview. J Nutr Sci.

[64] Cao Y, Zhuang M, Yang Y, Xie S, Cui J, Cao L (2014). Preliminary study of Quercetin affecting the hypothalamic-pituitary-gonadal Axis on rat endometriosis model. Evid Based Complement Alternat Med.

[65] Resende FA, de Oliveira APS, de Camargo MS, Vilegas W, Varanda EA (2013). Evaluation of Estrogenic Potential of Flavonoids Using a Recombinant Yeast Strain and MCF7/BUS Cell Proliferation Assay. Jeong J, editor. PLoS ONE.

[66] Hassoun E (1996). Cadmium-induced production of superoxide anion and nitric oxide, DNA single strand breaks and lactate dehydrogenase leakage in J774A.1 cell cultures. Toxicology..

[67] Liu F, Jan KY (2000). DNA damage in arsenite- and cadmium-treated bovine aortic endothelial cells. Free Radic Biol Med.

[68] Stohs SJ, Bagchi D, Hassoun E, Bagchi M (2000). Oxidative mechanisms in the toxicity of chromium and cadmium ions. J Environ Pathol Toxicol Oncol.

[69] Badisa VLD, Latinwo LM, Odewumi CO, Ikediobi CO, Badisa RB, Ayuk-Takem LT (2007). Mechanism of DNA damage by cadmium and interplay of antioxidant enzymes and agents. Environ Toxicol.

[70] Ciani F, Cocchia N, D’Angelo D, Tafuri S (2015). Influence of ROS on ovarian functions. New Discov Embryol.

[71] Ighodaro OM, Akinloye OA (2018). First line defence antioxidants-superoxide dismutase (SOD), catalase (CAT) and glutathione peroxidase (GPX): their fundamental role in the entire antioxidant defence grid. Alex J Med.

[72] Kohen R, Nyska A (2002). Oxidation of biological systems: oxidative stress phenomena, antioxidants, redox reactions, and methods for their quantification. Toxicol Pathol.

[73] Ahmed S, Sulaiman SA, Baig AA, Ibrahim M, Liaqat S, Fatima S (2018). Honey as a potential natural antioxidant medicine: an insight into its molecular mechanisms of action. Oxidative Med Cell Longev.

[74] Rice-Evans C, Miller N, Paganga G (1997). Antioxidant properties of phenolic compounds. Trends Plant Sci.

[75] Silva FAM, Borges F, Guimarães C, Lima JLFC, Matos C, Reis S (2000). Phenolic acids and derivatives: studies on the relationship among structure, radical scavenging activity, and physicochemical parameters. J Agric Food Chem.

[76] Ryan MJ, Dudash HJ, Docherty M, Geronilla KB, Baker BA, Haff GG (2010). Vitamin E and C supplementation reduces oxidative stress, improves antioxidant enzymes and positive muscle work in chronically loaded muscles of aged rats. Exp Gerontol.

